# Factors Associated with Do Not Resuscitate Status and Palliative Care in Hospitalized Patients: A National Inpatient Sample Analysis

**DOI:** 10.1089/pmr.2024.0030

**Published:** 2024-08-05

**Authors:** Jean-Sebastien Rachoin, Nicole Debski, Krystal Hunter, Elizabeth Cerceo

**Affiliations:** ^1^ Cooper University Healthcare, Cooper Medical School of Rowan University, Camden, New Jersey, USA.; ^2^ Cooper Medical School of Rowan University, Camden, New Jersey, USA.; ^3^ Cooper University Healthcare, Cooper Medical School of Rowan University, Camden, New Jersey, USA.

**Keywords:** DNR, ethnic disparities, gender disparities, health equity, palliative care

## Abstract

**Introduction:**

Patients from diverse sociocultural backgrounds and with differing medical conditions may have varying levels of acceptance of advanced care planning and palliative care.

**Methods:**

We performed a retrospective analysis of the National Inpatient Sample for patients discharged from January 1, 2016, to December 31, 2019, with conditions associated with frequently terminal conditions. We recorded demographic variables, do not resuscitate (DNR) status, and palliative care (PC) status and analyzed the associations between outcomes, mortality, and length of stay (LOS).

**Results:**

A total of 23,402,637 patient records were included in the study, of which 2% were DNR and PC, 5% were DNR only, and 1% was PC only. From 2016 to 2019, the percentage of patients with PC increased from 2.55% to 3.27% and DNR from 6.31% to 7.7%. Black patients were less likely to have DNR status (odds ratio [OR] 0.72 [0.71–0.72]) but had similar PC rates. Male patients were less likely to have a DNR order in place (OR 0.89 [0.89–0.89]) but more likely to be in PC (OR 1.05 [1.04–1.05]). The diagnoses with the highest association with DNR status were lung cancer (OR 4.1 [4.0–4.5]), pancreatic cancer (OR 4.6 [4.5–4.7]), and sepsis (OR 2.9 [2.9–2.9]) The diagnoses most associated with PC were lung cancer (OR 6.3 [6.2–6.4]), pancreatic cancer (OR 8.1 [7.1–8.3]), colon cancer (OR 4.9 [4.8–5.1]), and senile brain degeneration of the brain OR 6.5 [5.3–7.9]). Mortality and LOS decreased between 2016 and 2019, but hospital charges increased (*p* < 0.001). Black race and male gender were associated with higher inpatient mortality (OR 1.12 [1.12–1.14]), LOS, and hospital charges.

**Conclusion:**

In the United States, the proportion of hospitalized patients with DNR, PC, and DNR with PC increased from 2016 to 2019. Overall, inpatient mortality and LOS fell, but hospital charges per patient increased. Significant gender and ethnic differences emerged. Black patients and males were less likely to have DNR status and had higher inpatient mortality, LOS, and hospital charges.

## Introduction

The population in the United States is aging and experiencing more comorbidities and chronic illness burden.^1–4^ Specifically, there is an increased risk of chronic diseases such as dementia, heart disease, type 2 diabetes, arthritis, and cancer, with associated increased health care costs.^3,5–8^ This changing medical landscape represents a growing need to manage an older population with compounding medical burdens. Services such as palliative care (PC) and decisions regarding end-of-life care may become increasingly prominent focuses in health care delivery.^2,4^

PC, specialized medical care provided to individuals with serious illness that focuses on alleviating symptoms and enhancing quality of life, has become central in U.S. health care.^9,10^ It is often more cost-effective, significantly lowering the cost of end-of-life medical expenses^11,12^ and improving the quality of end-of-life care.^9,10,13^ The lower total health care costs were additionally not shifted to families, a finding particularly relevant to Americans with lower socioeconomic status.^9^

However, access to and utilization of PC resources and application of DNR orders are not consistent across demographic groups and among those with lower levels of health literacy, lower socioeconomic status, or less access to health care services and information.^10–14^ Such care inequities mirror other systemic injustices. As with other forms of health care, there are well-documented racial and ethnic disparities in access and quality of care. Some minority groups may hold specific cultural beliefs and preferences on the experience of pain, disability, serious illness, and death.^15–22^ Gender inequalities can additionally intersect with other social determinants of health to exacerbate situations of marginality.^23^ Thus, research to eliminate racial, ethnic, and gender disparities in PC is essential as intersectional identities may have additional care implications.

Given historical inequities of care, we hypothesized this may persist to end-of-life care as well. In this study, we analyze the diagnoses and demographic factors associated with PC or DNR status, because an understanding of the demographic factors connected to DNR status is essential to understanding the factors that influence end-of-life decisions. We further assess the outcomes associated with PC or DNR status, specifically length of stay (LOS), hospital charges, and mortality. We discuss these findings in the broader context of the current U.S. health care climate.

## Methods

### Study design

We performed a retrospective analysis of the National Inpatient Sample (NIS) for patients discharged from January 1, 2016, to December 31, 2019. The NIS represents the largest publicly available database of inpatient admissions in the United States, with over 7 million annual hospital stays.^24^ After being weighted, it estimates around 35 million hospitalizations nationally. The database is designed to represent hospitals and discharges nationally from the sampling frame required to net 20 percent of hospitals nationally. Developed through a federal–state–industry partnership sponsored by the Agency for Healthcare Research and Quality (AHRQ), Healthcare Cost and Utilization Project (HCUP) data inform national, state, and community decision making. We chose this timeframe to avoid changes in international classification of diseases (ICD) coding that occurred in 2015 and the impact of the pandemic.

### Diagnoses of interest

We examined all records of hospitalized patients. We only included patients with the following ICD10 discharge diagnoses: Alzheimer’s disease (ICD10 G30.9, 3G0.1), chronic obstructive pulmonary disease (COPD) (J44.9, J44.1), chronic heart failure (CHF) (I50.9, I11.0, I13.0), senile degeneration of brain (G31.1) lung malignancy (C34.90), Parkinson’s disease (G20), cardiovascular disease (I125.10), prostate cancer (C61), colon cancer (C18.9), end-stage renal disease (N18.6), pancreatic cancer (C25.9), and sepsis (A41.9). These conditions were selected based on a presumption of a high likelihood of inpatient mortality resulting from the disease and on leading causes of death in the United States.^25^ We excluded diabetes because it may be a comorbidity in a more serious reason for admission, and mortality may not be as imminent as the other conditions. Diagnoses could be added to the hospital record throughout the hospitalization.

### Variables and outcomes

We recorded demographic variables (age, gender, and race), DNR status (ICD10 Z66), and PC encounter (ICD10 Z51.5) and then divided the population into subgroups based on gender, race, and age by deciles.

The outcomes of interest were inpatient mortality, hospital LOS, and hospital charges. Analyses were adjusted for comorbid conditions using the Charlson index. This index includes myocardial infarction, CHF, PVD, CVA or transient ischemic attack, dementia, COPD, connective tissue disease, peptic ulcer disease, liver disease, diabetes mellitus, hemiplegia, moderate-to-severe chronic kidney disease, solid tumor, leukemia, and AIDS. The NIS provides categories for calculating the Charlson comorbidity index, and comorbidities can be reported separately outside the index.

### Statistical methods

The HCUP-NIS data were uploaded from the website (https://hcup-us.ahrq.gov/nisoverview.jsp). The website has the data in the file formats SPSS, Stata, and SAS for all years from 1997 to 2021. We used the SAS file format.

Two load programs read the data into SAS and prepare it for analysis. We first excluded those who were younger than 18 years. We also deleted missing or nonvalid data. Although the NIS file has basic demographic data, it does not report prior medical history, PC status, or DNR status. To capture that information, we had to compile a listing of ICD10 codes used to create these data elements. We transferred the data from SAS (SAS, Cary, NC), and all statistical analyses were performed using SPSS 27 (IBM, Armonk, NY).

We present categorical variables as frequencies and percentages. Continuous variables with minimal skew (indicating a normal distribution) are presented as means and standard deviations. Continuous variables that have significant skew are presented with medians and interquartile ranges. We analyzed trends using the Cochran–Armitage Test, which was also used to analyze trends in all patients and subgroups. Logistic regression models were applied to PC, DNR, and mortality outcomes. LOS and total charges were logarithmically transformed to run linear regression with these as dependent variables. We entered year, gender, age, insurance, race, Charlson, and all diagnoses of interest into the model.

Cooper University Health Care Institutional Review Board deemed this study to be exempt. We followed the Strengthening the Reporting of Observational Studies in Epidemiology (STROBE) reporting guidelines for cohort studies. All analyses were performed using SPSS 27 (IBM).

## Results

A total of 23,402,637 patient records were included in the study, of which 2% were both DNR and PC, 5% were DNR only, and 1% was PC only.

The average age was highest in patients with a DNR order (78.8 [11.9]) compared with PC and DNR order (75.3 [15.4]), PC only (70 [15.3]), and no PC/no DNR (56.5 [20]). More males had PC orders (49.2%), PC and DNR (47.3%), no PC/no DNR (42.3%), and DNR only (41.2%). Black race was lower in the groups PC (14.9%), DNR (7.4%), and PC and DNR (11.2%) compared with no PC/no DNR (15.7%). Medicare was the predominant insurance in all groups: PC (63.9%), DNR (85.7%), PC and DNR (73.8%), and no PC/no DNR (45%). Mortality was 0.8% in the group with no PC/no DNR, 9.3% in DNR only, 27.4% in PC only, and 39.2% in PC and DNR. The LOS and charges were highest in the PC group, followed by PC and DNR, DNR only, and no PC/no DNR (Table 1).

**Table 1.** Demographic Variables by Groups

	PC	DNR	PC and DNR	No PC/no DNR	All patients
Number	214,730	1,174,338	482,982	21,530,587	2,3402,637
Age (Years)^a^	70 (15.3)	78.8 (11.9)	75.3 (15.4)	56.5 (20)	57.7 (19.4)
Male gender	105,587 (1.1%)	483,770 (4.9%)	228,555 (2.3%)	9,114,298 (91.8%)	9,932,210 (42.4%)
Race					
Black	32,086 (0.9%)	86,777 (2.4%)	54,235 (1.5%)	3,380,364 (95.1%)	3,553,462 (15.1%)
White	152,646 (1%)	962,818 (6.1%)	366,296 (2.3%)	14,268,930 (90.6%)	15,750,690 (67.3%)
Hispanic	17,940 (0.7%)	67,869 (2.6%)	34,546 (1.3%)	2,484,635 (95.4%)	2,604,990 (11.1%)
Asian	5,740 (0.9%)	28,348 (2.4%)	13,977 (2.9%)	598,159 (2.8%)	646,224 (2.8%)
Native Americans	1,279 (0.9%)	5,519 (3.7%)	2,338 (1.6%)	140,361 (93.9%)	149,497 (0.6%)
Other	5,039 (0.7%)	23,007 (3.3%)	11,590 (1.7%)	657,868 (94.3%)	697,504 (3%)
Insurance					
Medicare	137,296 (1.2%)	1,006,041 (9%)	356,512 (3.2%)	9,697,767 (86.6%)	11,197,616 (47.9%)
Medicaid	23,475 (0.5%)	50,966 (1.2%)	33,001 (0.8%)	4,202,574(97.5%)	4,310,016 (18.4%)
Private	38,869 (0.6%)	88,265 (1.4%)	65,237 (1.1%)	6,011,510 (96.9%)	6,203,881 (26.5%)
Self-pay	4,457 (0.5%)	11,164 (1.2%)	8,053 (0.9%)	923,997 (97.5%)	947,671 (4.1%)
No charge	310 (0.4%)	701 (0.8%)	559 (0.7%)	81,488 (0.45)	83,058 (98.1%)
Other	10,323 (1.6%)	17,201 (2.6%)	19,620 (3%)	613,251 (92.9%)	660,395 (2.8%)
Regions					
Northeast	40,843 (0.9%)	242,649 (5.5%)	86,687 (2%)	4,075,918 (91.7%)	4,405,295 (18.8%)
Midwest	46,894 (0.9%)	284,399 (5.6%)	114,359 (2.3%)	4,614,964 (91.2%)	5,060,616 (21.6%)
South	85,156 (0.9%)	378,084 (4%)	183,743 (2%)	8,728,859 (93.1%)	9,375,842 (40.1%)
West	41,837 (0.9%)	269,206 (6%)	98,193 (2.2%)	4,110,846 (91%)	4,520,082 (19.3%)
Mortality	58,681 (11.3%)	108,631 (21%)	189,567 (36.5%)	162,120 (31.2%)	518,999 (2.2%)
Length of stay^b^	6 [3–11]	4 [3–7]	5 [2–9]	3 [2–5]	3.1 [2–5.2]
Charges (/$1,000)^b^	50 [23–109]	34 [19–64]	44 [21–94]	31 [17–61]	31.6 [17.2–62.3]

^a^ Average (± standard deviation).

^b^ Median [interquartile range].

PC, palliative care.

Table 2 presents the number of patients by year, per diagnosis, based on the subgroups. The proportions and number of patients in DNR, PC, and DNR and PC increased from 2016 to 2019. The most common diagnoses were sepsis, COPD, and CHF in all categories.

**Table 2.** Discharge Year and Diagnosis by Groups

	PC	DNR	PC and DNR	No PC/no DNR	All patients
Discharge year					
2016	52,300 (0.9%)	262,455 (4.6%)	101,028 (1.75%)	5,347,575 (92.8%)	5,763,358 (24.6%)
2017	53,718 (0.9%)	289,411 (4.9%)	116,627 (2%)	5,397,629 (92.2%)	5,857,385 (25%)
2018	53,291 (0.9%)	306,260 (5.2%)	127,956 (2.2%)	5,401,243 (91.7%)	5,888,750 (25.2%)
2019	55,421 (0.9%)	316,212 (5.4%)	137,371 (2.3%)	5,384,140 (91.4%)	5,893,144 (25.2%)
Diagnoses					
Alzheimer	7,282 (2.5%)	68,302 (23.5%)	25,628 (8.8%)	190,027 (62.3%)	291,239 (1.24%)
Senile degeneration of the brain	35 (7.5%)	76 (16.3%)	109 (23.3%)	247 (52.9%)	467 (0.002%)
Parkinson	4397 (1.6%)	39,959 (14.8%)	13,637 (5.1%)	211,510 (78.5%)	269,503 (1.15%)
CVD	4736 (1.8%)	24,229 (9.2%)	13,055 (5%)	220,629 (84%)	262,649 (1.12%)
COPD	45,133 (1.3%)	282,491 (8.3%)	102,170 (3%)	2,989,334 (87.4%)	3,419,128 (14.6%)
CHF	46,041 (1.6%)	320,477 (10.8%)	121,078 (4.1%)	2,486,010 (83.6%)	2,973,606 (12.7%)
ESRD	12,565 (1.5%)	43,416 (5.3%)	21,344 (2.6%)	739,349 (90.5%)	816,674 (3.5%)
Sepsis	40,862 (2.7%)	173,124 (11.2%)	144,155 (9.4%)	1,183,603 (76.8%)	1,541,744 (6.6%)
Lung m alignancy	7266 (6%)	16,595 (13.7%)	15,869 (13.1%)	81,686 (67.3%)	121,416 (0.5%)
Prostate cancer	3799 (2.5%)	12,796 (8.3%)	7535 (4.9%)	130,701 (84.4%)	154,831 (0.7%)
Colon cancer	2693 (5.4%)	4899 (9.9%)	4842 (9.8%)	37,235 (75%)	49,669 (0.2%)
Pancreatic cancer	3523 (7.4%)	5739 (12%)	6845 (14.4%)	31,544 (66.2%)	47,651 (0.2%)

COPD, chronic obstructive pulmonary disease; CHF, chronic heart failure; CVD, cardiovascular disease; ESRD, end-stage renal disease.

### Trends over time in the univariate analysis

**FIG. 1.**
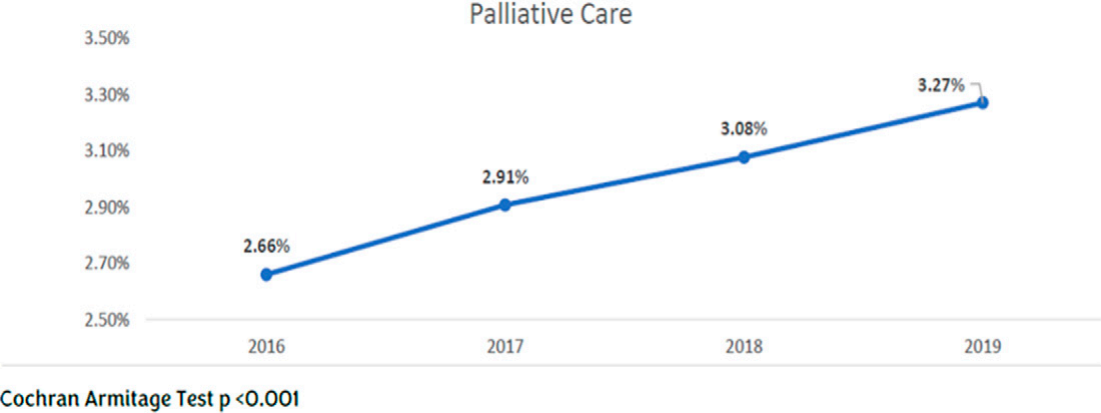
Palliative care rates from 2016 to 2019 in hospitalized patients.

**FIG. 2.**
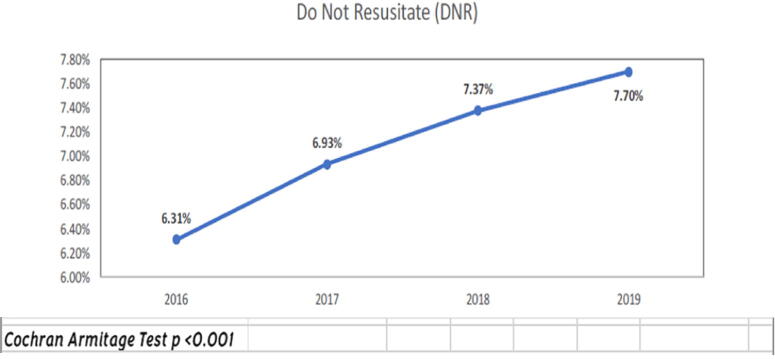
DNR status rates from 2016to 2019 in hospitalized patients.

From 2016 to 2019, the percentage of patients with PC increased from 2.55% to 3.27% (*p* < 0.001) and DNR from 6.31% to 7.7% (*p* < 0.001). We included any patient with a DNR order was linked to the DNR category and any patient with PC was in the PC category (Fig. 1 and Fig. 2).

In the subgroup analysis, this increase was consistent for DNR and PC among all age groups, except in the <50-year age-group. When looking at race categories, there was a statistical increase in PC rates in all subgroups, except for Native Americans and others. The change, however, was relatively small. DNR status increased in White, Hispanic, and Asian patients but not in Blacks, Native Americans, and others. Both males and females had increased rates of DNR status over time, but the increase was only seen in females for PC. All geographic regions across the United States experienced increased DNR; for PC status, only the Midwest and West did (Supplementary Table S1) (Supplementary Figures 1–8).

### Likelihood of PC and DNR in the multivariate analysis

Using logistic regression, Black patients (compared with Whites) were less likely to have DNR status (adjusted odds ratio [OR] 0.72 [0.71–0.72]) but had similar rates of PC. Male patients, compared with females, were less likely to have a DNR order in place (adjusted OR 0.89 [0.89–0.89]) but more likely to be in PC (adjusted OR 1.05 [1.04–1.05]).

The diagnoses with the highest association with DNR status were lung cancer (vs. no lung cancer) (adjusted OR 4.1 [4.0–4.5]), pancreatic cancer (vs. no pancreatic cancer) (adjusted OR 4.6 [4.5–4.7]), and sepsis (vs. no sepsis) (adjusted OR 2.9 [2.9–2.9]) (all *p* < 0.001). The diagnoses most associated with PC were lung cancer (vs. no lung cancer) (adjusted OR 6.3 [6.2–6.4]), pancreatic cancer (adjusted OR 8.1 [7.1–8.3]), colon cancer (adjusted OR 4.9 [4.8–5.1]), and senile brain degeneration of the brain (adjusted OR 6.5 [5.3–7.9]). (Supplementary Table S2).

### Outcomes mortality, LOS, and charges

Mortality and LOS decreased between 2016 and 2019, but hospital charges increased (*p* < 0.001). Black race (compared with White) (adjusted OR 1.12 [1.12–1.14]) and male gender (compared with female) (adjusted OR 1.38 [1.37–1.39] *p* < 0.001) were associated with higher inpatient mortality, LOS, and hospital charges (Supplementary Table S3). Compared with patients without PC or DNR, patients with DNR only, PC only, and DNR and PC had higher inpatient mortality (adjusted OR 9.8 [9.7–9.9]), 35.8 [35.4–36.2], and 60.6 [60.1–61.1], respectively), LOS, and charges (all *p* < 0.001), except patients with DNR only had lower charges (all *p* < 0.001).

## Discussion

Early integration of PC with a focus on symptom management and quality of life provides significant benefits at the end of life.^26^ However, the benefits of effective end-of-life care are not proportionately distributed. As with other aspects of health care disparities, gender and minority status have implications for clinical practice and patient experience.^12,14^ Much of the recent research has not been conducted at the population level, but rather with smaller groups of individuals. This nationwide survey of end-of-life practices permits broad generalization, which may help inform more equitable end-of-life practices.

### Gender differences

One framework to interpret the results is through a gender lens, although other perspectives may be valid. Gender differences in inpatient care and decision making, while they are culturally contingent,^27–31^ also reflect a pervasive belief that women are natural caregivers and men must pursue aggressive treament.^30,32,33^ These gender constructs disadvantage both sexes. For women, gender impacts caregiving roles,^33,34^ with an unspoken expectation that women, even if older or ill, will provide end-of-life care, despite experiencing considerable burden. The finding of fewer female participants in PC mirrors existing cultural norms in which ∼75% of caregivers identify as female, with women spending 50% more time providing care.^35,36^ Much end-of-life care occurs in the community setting, which may place more burden on women caregivers and result in less available support when women become ill.^34,37^ Health care professionals may unconsciously perpetuate these stereotypes by providing more resources for male caregivers, considering them as less “natural” in the role.^32,38^ Women also tend to have longer life expectancies with the unfortunate corollaries of a lack of support from death of spouses and other supportive individuals, depletion of financial resources (exacerbated by lower earning potential throughout their life span), and a greater burden of symptoms and suffering.^32,39,40^ As a result, the option to choose more care in the home may disproportionately be less available to women.

Women were also found to have greater acceptance of DNR status,^40^ which may be considered in the context of societal expectations where women’s decisions should align with creating the least burden on the family. In support of this, women have shorter hospitalizations and are more likely to be cared for in a nursing facility than at home.^23,32,39,40^ In addition, women tend to prioritize continuity with a primary care physician. In contrast, men seek more aggressive care from specialists, which may align with the lower rates of DNR status seen in male patients.^32,40,41^ We also found that fewer women die in the hospital, which may suggest less aggressive care as they would then die at home, nursing facilities, or elsewhere.

Men too suffer from imposed gender constructs in that vulnerability may be seen as a weakness and strength prioritized.^29–33^ In our study, male patients were significantly less likely to be DNR but more likely to be engaged in PC, consistent with prior studies demonstrating analogous demographic patterns in DNR status.^42,43^ Although it is difficult to make sweeping assumptions of motivations based on a database, it suggests that men may be more reluctant to relinquish control of code status, opting for more aggressive care, but philosophically consent to PC and have the resources (both family and financial) to accept PC.

### Racial differences

The percentage of patients in our study receiving PC has consistently increased from 2.55% in 2016 to 3.27% in 2019, along with those choosing to be DNR (6.3% in 2016 to 7.7% in 2019). This may be due to improved communication with patients or increased patient knowledge but is not uniform among different demographics. However, Black patients were still less likely to be DNR than other races, a finding consistent with prior studies, where DNR orders were documented nearly half as often for Black patients as for their white counterparts.^42,43^

These observations prompt this question: What factors contribute to variations in treatment choices based on race? A socioecological framework considers the various interacting structures and oppressive practices of systemic racism that create barriers, influencing health outcomes.^44^ For example, centuries of racial injustices may engender suspicion of institutional health care such that minority patients may feel inclined to select more aggressive treatment options, driven by the concern that an alternative approach could result in suboptimal medical care.^14^ Personal ideals, familial preferences, and cultural or religious values may play a role.^43,45^ A patient’s spiritual and religious beliefs may not align with health care’s conception of end-of-life care.^44,46,47^ However, it is important to consider the social inequities that are likely contributing to what occurs throughout the lifespan and not only at the end of life. These disparities are partly attributable to differences in access to high-quality health care,^24^ but even when care is accessible, differences in quality of care may be apparent.

Prior studies have demonstrated that physicians have fewer end-of-life discussions with Black patients despite similar desires for such conversations across different racial groups.^43^ The absence of these discussions represents a lost chance for individuals to understand available options for end-of-life care. It limits their ability to make decisions in alignment with their values. Racial and ethnic minorities less frequently complete advanced directives and engage in advanced care planning, which may reflect on the quality of care provided.^14^ Persons from racial minorities are more likely to receive aggressive treatments, including mechanical ventilation and cardiopulmonary resuscitation (CPR), which are associated with a lower quality of life and higher health care expenditures.^14,48,49^ Racial minority patients more frequently are unsatisfied with their care at the end of life and receive goal-discordant care^12,47,49,50^ while simultaneously having more pain and symptoms.^18^

### Cost trends

Trends in DNR status and PC are against the backdrop of overall trends in health care such as inpatient cost, mortality, and LOS. Our study revealed a decline in inpatient mortality rates, which may coincide with increased PC utilization. The concomitant increase in per-patient hospital charges may align with current demographic shifts in the United States, where people are living longer than before but are also facing elevated levels of illness burden.^4,5^ Heightened morbidity, influenced by both the extended life expectancy and factors like lifestyle choices, stress, and environmental elements, may play a role in the observed rise in health care costs but is an area that deserves additional scrutiny. As the U.S. population ages and health care funding becomes increasingly stretched, cost-effective and congruent care will be ever more important,^51^ especially since CPR frequently diverges from patients’ true preferences.^43,52,53^ Health care costs have increased nationally, but the disproportionate expenditure on Black or male patients suggests true differences in the care delivered. Some of this expense may be due to the increased LOS witnessed in these populations, but it is possible that procedures, testing, and consults may contribute to the expense as well. The nature of the database does not permit cost extraction for finer detail of expenses. More expensive care such as the intensity in care given to some patients over others may represent another form of inequity. Of course, more expensive care is not always better care and these findings should prompt reflection at the individual patient level.

### Overall trends

A small number of patients in this overall sample of hospitalized patients had both DNR and PC established (2%), with 5% being DNR only and 1% being in PC only. Given the increasing illness and comorbidity of the U.S. population, the low rates are notable. This could either imply that individuals who are in PC are not entering the hospital setting, which would align with care goals in that scenario, or that many more patients would benefit from PC in the hospital but are not yet receiving it. However, proportionally, more adults are adopting PC options in the hospitalized population. One study revealed that nearly one-third of patients documented as full code would have preferred DNR if they had been adequately informed about the option.^53^ Hence, although the overall rise in our sample of patients opting for DNR may indicate a broader societal comprehension of code status and a growing trend of patients exercising autonomy in medical decision making, the observed racial and gender disparities highlight the need for increased efforts to address equity within this domain. In addition, those with PC and DNR status were significantly more likely to be Medicare beneficiaries, an expected association with end-of-life care.

Limitations of our study include those inherent to database research, such as an inability to ascertain nuance and fine descriptors of patients, including sex and gender, which have historically been collapsed into binaries in the electronic medical record. However, this does not adequately reflect patients’ experiences or identities. A similar lack of nuance results from race and ethnicity descriptors as limited choices can collapse a patient’s identity down to categories that ill-define their cultural experiences. The NIS database sample is drawn from the sampling frame consisting of discharge data submitted by HCUP Partners, which are statewide data organizations that agree to participate in the NIS. Each entry in the database is an individual encounter, but no patient identifier is provided, so we were unable to assess multiple admissions for the same patient. The complexity of motivations that influence these critical decisions could be better assessed with interviews and focus groups among various constituent groups. Owing to the nature of the data collected, we could not account for patients with multiple hospital admissions and selected conditions with high likelihood of inpatient mortality. Not all conditions were covered, but, = rather, representative and highly fatal conditions were chosen. The ICD10 code Z51.5 does not specify who conducts the PC encounter, which could be led by a PC professional, a primary physician, or another health care professional. It similarly does not permit representation of the qualitative aspects of PC, specifically the depth and subtlety of these interactions. Finally, there is a risk of overcoding or undercoding, but, because the dataset lacks access to a “gold standard” for PC episodes, conclusions cannot be drawn as to the integrity of the data.

## Conclusion

Despite the limitations, our study confirms in a large database that distinctive racial and gender patterns emerge in the context of PC and DNR status. These findings are coupled with an escalation in health care expenses. Meeting patients’ needs with attention to health equity and minimization of disparities is paramount in effective care delivery across populations. It not only has patient outcome consequences but also has financial implications as end-of-life care tends to be the most expensive across the lifespan, often with outcomes not congruent with patients’ ultimate wishes. Clinicians should consider the possibility of health inequities as contributing to end-of-life choices, using an individual’s background to inform sensitive conversations around shared decision making.

## Abbreviations Used

CHF: Chronic Heart failure
CKD: Chronic Kidney disease
COPD: chronic Obstructive Pulmonary Disease
CVD: Cardiovascular disease
DNR: Do Not Resuscitate
ESRD: End-Stage Renal disease
ICD: International Classification of Diseases
TIA: Transient Ischemic Attack
