# Supplementary material for: Factors Associated with Do Not Resuscitate Status and Palliative Care in Hospitalized Patients: A National Inpatient Sample Analysis
Source: Palliat Med Rep. 2024 Aug 5;5(1):331–9. doi: 10.1089/pmr.2024.0030 (PMC11319862; doi:10.1089/pmr.2024.0030)
Supplement: Supplemental Figure S1 [file pmr.2024.0030_supplementalmaterialdnrpaper.pdf]

Supplemental Material

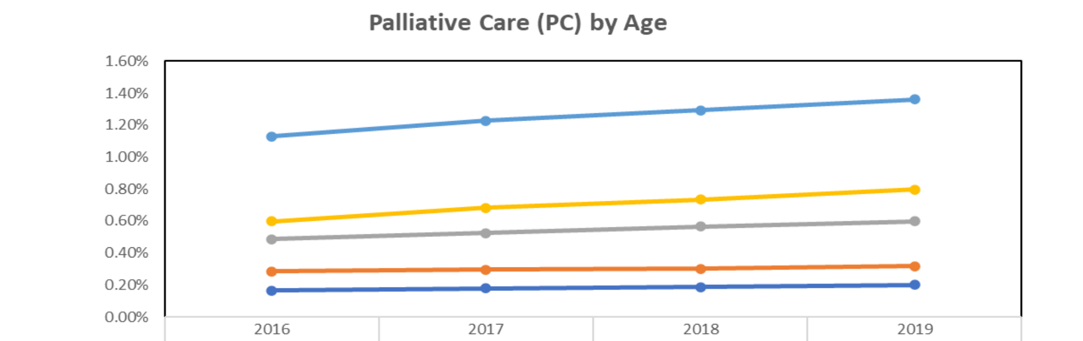

	2016	2017	2018	2019
Age < 50	0.17%	0.18%	0.19%	0.20%
Age 50 - 59	0.28%	0.29%	0.30%	0.32%
Age 60 - 69	0.48%	0.53%	0.56%	0.60%
Age 70 - 79	0.60%	0.68%	0.73%	0.80%
Age >= 80	1.13%	1.23%	1.29%	1.36%

	(Cochran Armitage)								
Age <50	0.166	Age 50 - 59	<0.001	Age 60 - 69	<0.001	Age 70 - 79	<0.001	Age >= 80	<0.001

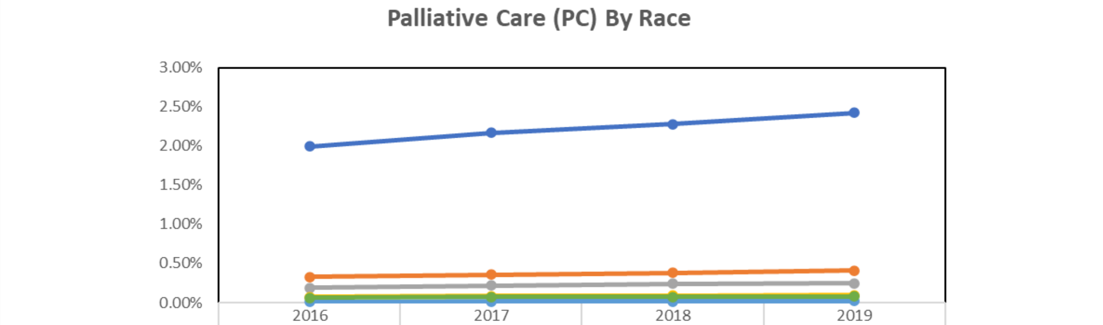

	2016	2017	2018	2019
White	2.00%	2.17%	2.28%	2.42%
Black	0.33%	0.36%	0.38%	0.41%
Hispanic	0.19%	0.22%	0.24%	0.25%
Asian	0.07%	0.08%	0.09%	0.10%
Native American	0.01%	0.01%	0.02%	0.02%
Other Races	0.06%	0.07%	0.07%	0.08%

	P-Value (Cochran										
White	<0.001	Black	0.032	Hispanic	<0.001	Asian	<0.001	Native American	0.277	Other Races	0.694

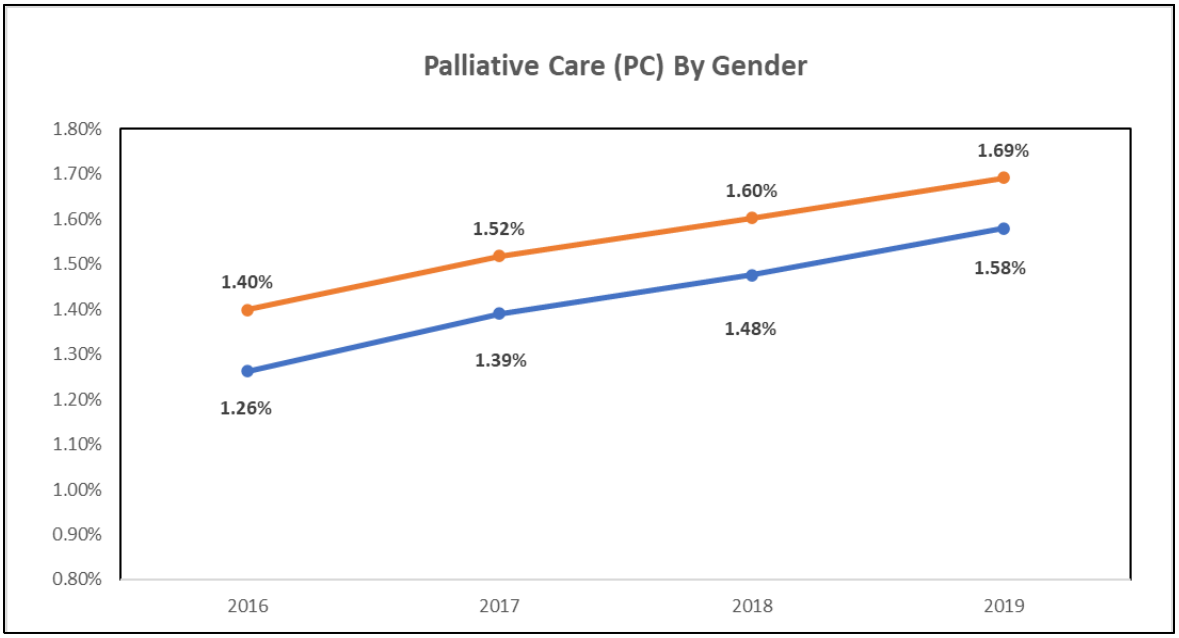

	P-Value (Cochran		
Male	0.290	Female	<0.001

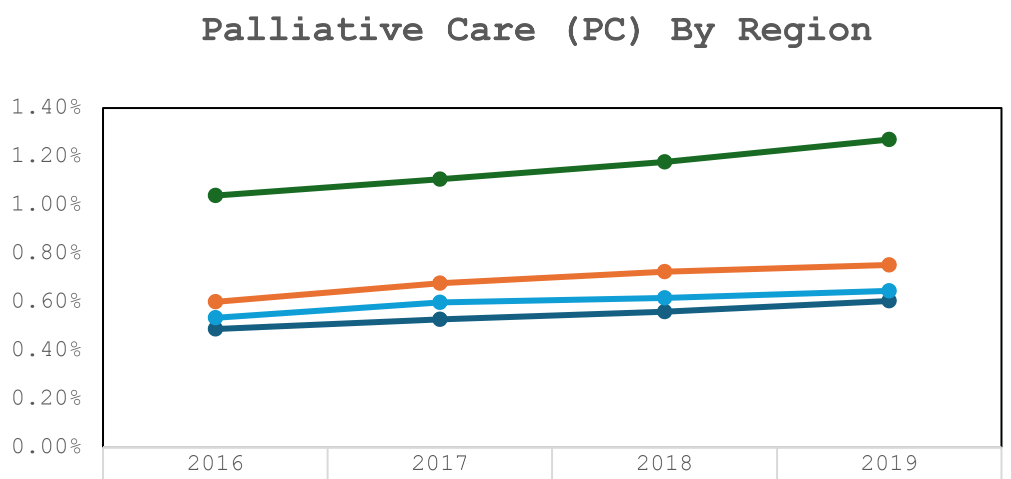

	2016	2017	2018	2019
NE	0.49%	0.53%	0.56%	0.60%
MW	0.60%	0.68%	0.72%	0.75%
S	1.04%	1.11%	1.18%	1.27%
W	0.53%	0.60%	0.62%	0.65%

	P-Value (Cochran						
NE	0.264	MW	0.002	S	0.716	W	<0.001

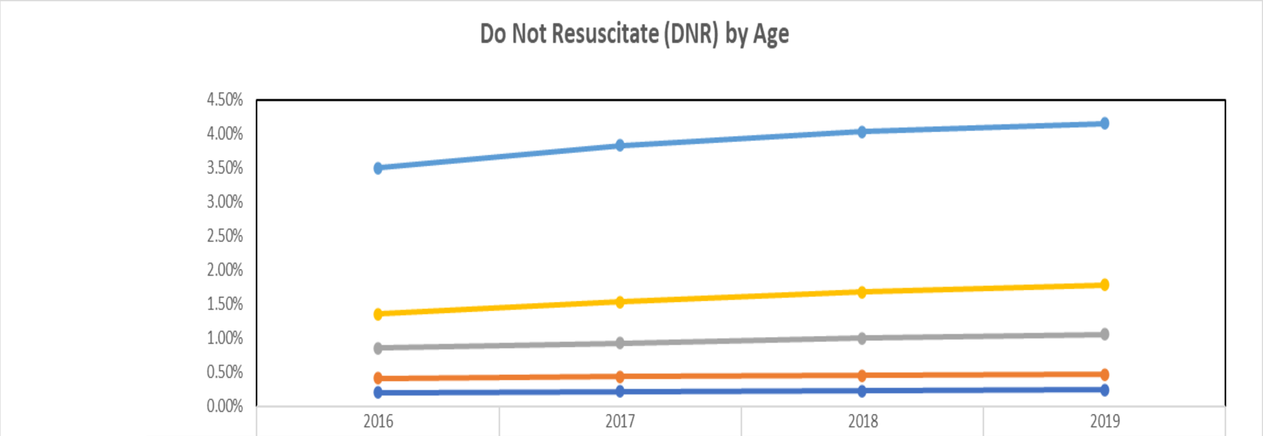

	2016	2017	2018	2019
Age < 50	0.20%	0.21%	0.23%	0.24%
Age 50 - 59	0.41%	0.43%	0.45%	0.46%
Age 60 - 69	0.85%	0.92%	0.99%	1.05%
Age 70 - 79	1.35%	1.53%	1.67%	1.78%
Age >= 80	3.50%	3.83%	4.03%	4.16%

	P- Value (Cochran Armitage)								
Age <50	0.722	Age 50 - 59	<0.001	Age 60 - 69	<0.001	Age 70 - 79	<0.001	Age >= 80	<0.001

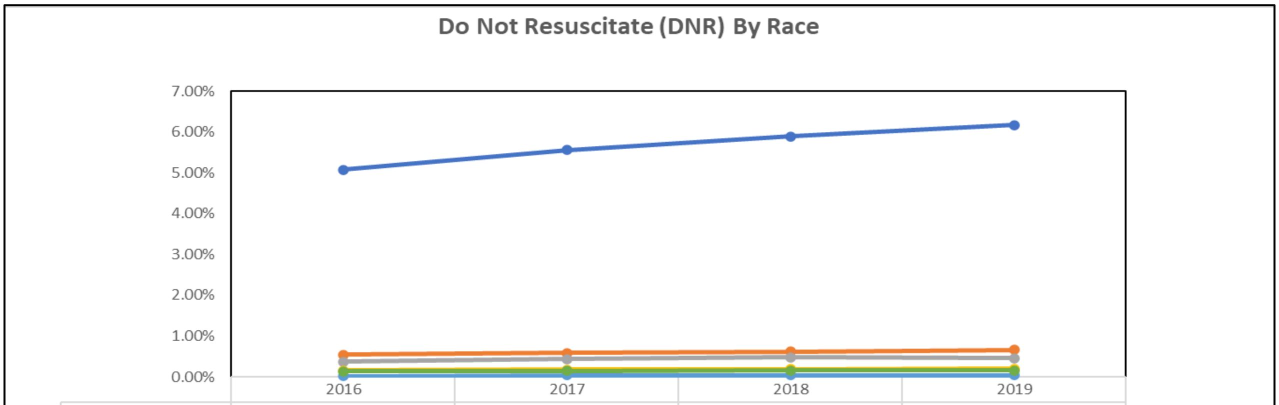

	2016	2017	2018	2019
White	5.07%	5.56%	5.89%	6.18%
Black	0.54%	0.59%	0.62%	0.66%
Hispanic	0.37%	0.43%	0.48%	0.46%
Asian	0.15%	0.18%	0.19%	0.20%
Native American	0.03%	0.03%	0.03%	0.04%
Other Races	0.13%	0.15%	0.16%	0.16%

	P- Value (Cochran Armitage)										
White	0.003	Black	0.308	Hispanic	0.039	Asian	<0.001	Native American	0.077	Other Races	0.867

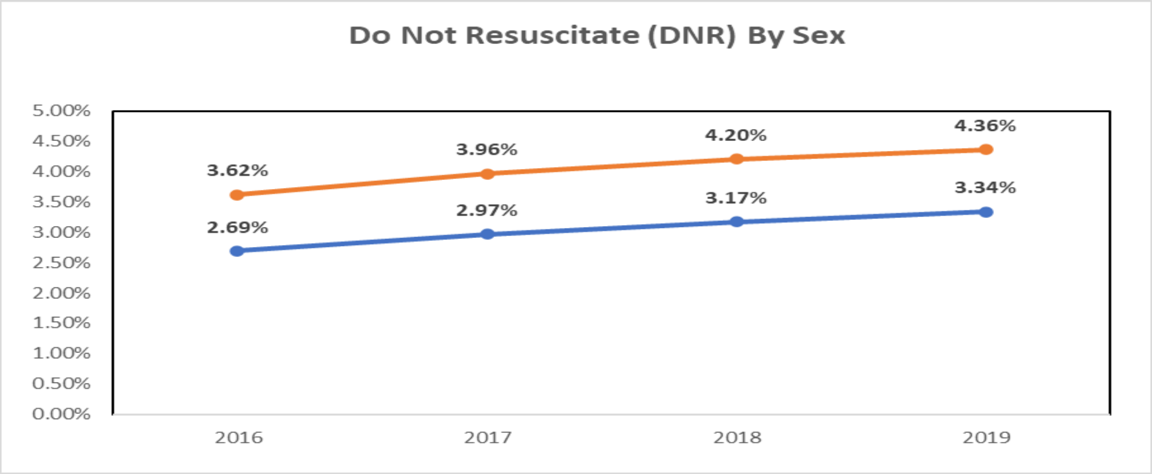

	P-Value (Cochran Armitage)		
Male	<0.001	Female	<0.001

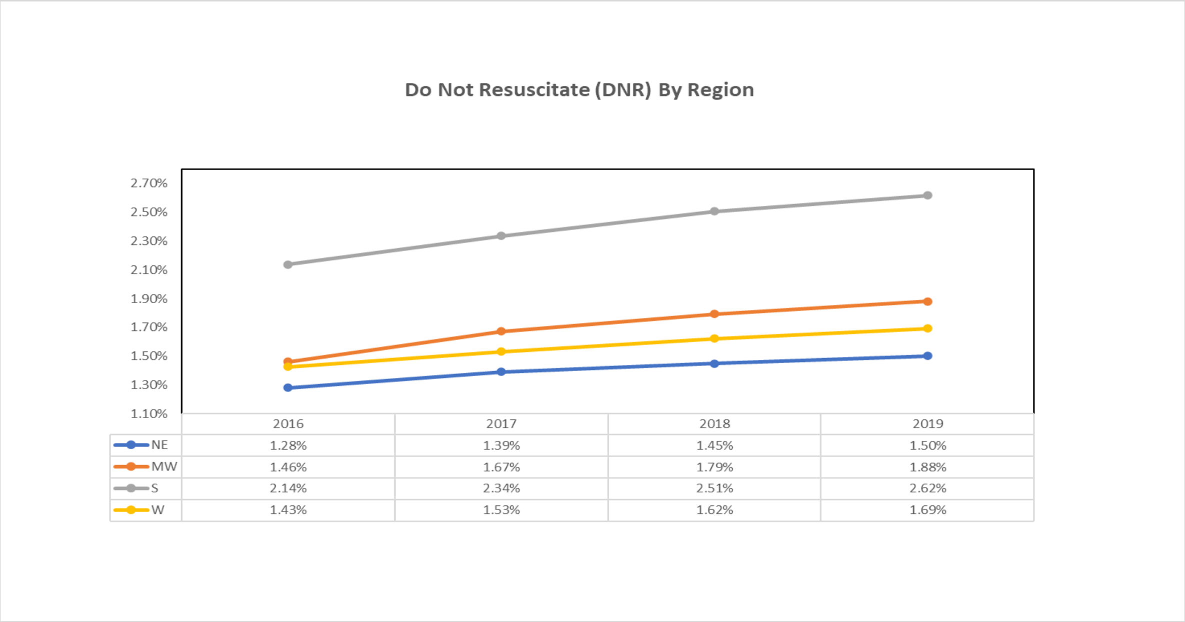

	P-Value (Cochran						
NE	<0.001	MW	<0.001	S	0.018	W	<0.001

Logistic regression , outcome Palliative care

**Logistic Regression Outcome = Palliative Care (PC)**	**B**	**Sig.**	**Exp(B)**	**95% C.I.for EXP(B)**	
				**Lower**	**Upper**
YEAR					
2016 v 2017	0.045	<0.001	1.046	1.039	1.054
2016 v 2018	0.093	<0.001	1.098	1.090	1.106
2016 v 2019	0.146	<0.001	1.157	1.149	1.165
AGE	0.051	<0.001	1.053	1.052	1.053
Gender - Male	0.048	<0.001	1.049	1.044	1.054
Insurance					
Medicare vs Medicaid	0.448	<0.001	1.565	1.549	1.581
Medicare vs Private	0.318	<0.001	1.375	1.364	1.386
Medicare vs Self-Pay	0.312	<0.001	1.366	1.340	1.392
Medicare vs No Charge	0.095	0.006	1.100	1.028	1.177
Medicare vs Other Insurance	1.013	<0.001	2.754	2.719	2.789
RACE					
Black v White	0.007	0.094	1.007	0.999	1.014
Hispanic v White	-0.148	<0.001	0.862	0.854	0.871
Asian v White	0.013	0.092	1.013	0.998	1.029
Native American v White	0.028	0.110	1.028	0.994	1.064
Other Races v White	-0.062	<0.001	0.940	0.925	0.955
CCI	0.033	<0.001	1.034	1.031	1.037
REGION					
NE vs MW	0.135	0.001	1.145	1.136	1.154
NE vs S	0.053	0.039	1.055	1.047	1.062
NE vs W	0.130	<0.001	1.138	1.129	1.147
Alzheimer's	0.626	<0.001	1.869	1.847	1.893
CHF	0.222	<0.001	1.249	1.240	1.257
Colon Cancer	1.604	<0.001	4.974	4.847	5.106
COPD	0.012	0.001	1.012	1.005	1.019
CVD	0.591	<0.001	1.807	1.778	1.836
Senile degeneration of the brain	1.866	<0.001	6.464	5.269	7.929
ESRD	0.501	<0.001	1.650	1.631	1.669
Lung Cancer	1.841	<0.001	6.301	6.205	6.398
Pancreatic Cancer	2.088	<0.001	8.069	7.885	8.257
Parkinson's Disease	0.241	<0.001	1.273	1.253	1.293
Prostate Cancer	0.545	<0.001	1.725	1.691	1.761
Sepsis	1.273	<0.001	3.571	3.549	3.593
Constant	-7.643	<0.001	0.000		

Logistic Regression, Outcomes DNR

Logistic Regression Outcome = Do Not Resuscitate (DNR)	B	Sig.	Exp(B)	95% C.I.for EXP(B)	
				Lower	Upper
YEAR					
2016 v 2017	0.053	<0.001	1.054	1.049	1.059
2016 v 2018	0.117	<0.001	1.124	1.119	1.130
2016 v 2019	0.159	<0.001	1.173	1.167	1.179
AGE	0.078	<0.001	1.081	1.081	1.082
Gender - Male	-0.118	<0.001	0.888	0.885	0.892
Insurance					
Medicare vs Medicaid	0.312	<0.001	1.366	1.355	1.378
Medicare vs Private	-0.025	<0.001	0.975	0.969	0.981
Medicare vs Self-Pay	0.204	<0.001	1.226	1.208	1.245
Medicare vs No Charge	-0.010	0.729	0.990	0.935	1.048
Medicare vs Other Insurance	0.404	<0.001	1.498	1.481	1.516
RACE					
Black v White	-0.332	<0.001	0.718	0.713	0.722
Hispanic v White	-0.371	<0.001	0.690	0.685	0.695
Asian v White	-0.210	<0.001	0.811	0.802	0.820
Native American v White	0.001	0.963	1.001	0.976	1.025
Other Races v White	-0.224	<0.001	0.799	0.790	0.808
CCI	0.001	0.459	1.001	0.999	1.003
REGION					
NE vs MW	0.091	<0.001	1.095	1.089	1.101
NE vs S	-0.134	<0.001	0.875	0.870	0.879
NE vs W	0.245	<0.001	1.277	1.270	1.284
Alzheimer's	0.801	<0.001	2.227	2.208	2.246
CHF	0.295	<0.001	1.343	1.337	1.350
Colon Cancer	1.067	<0.001	2.907	2.837	2.979
COPD	0.163	<0.001	1.176	1.171	1.182
CVD	0.418	<0.001	1.520	1.502	1.538
Senile degeneration of the brain	1.123	0.082	3.073	2.527	3.738
ESRD	0.376	<0.001	1.456	1.444	1.469
Lung Cancer	1.411	<0.001	4.098	4.042	4.156
Pancreatic Cancer	1.522	<0.001	4.581	4.481	4.684
Parkinson's Disease	0.429	<0.001	1.536	1.521	1.552
Prostate Cancer	0.306	<0.001	1.358	1.337	1.380
Sepsis	1.055	<0.001	2.873	2.859	2.887
Constant	-8.296	<0.001	0.000		

Outcome Mortality

**Logistic Regression Outcome = Mortality**	**B**	**Sig.**	**Exp(B)**	**95% C.I.for EXP(B) Lower**	**Upper**
YEAR					
2016 v 2017	-0.097	<0.001	0.908	0.900	0.916
2016 v 2018	-0.157	<0.001	0.855	0.847	0.862
2016 v 2019	-0.210	<0.001	0.811	0.804	0.818
AGE	0.010	<0.001	1.010	1.009	1.010
Gender - Male	0.323	<0.001	1.381	1.372	1.389
Insurance					
Medicare vs Medicaid	0.205	<0.001	1.228	1.213	1.244
Medicare vs Private	0.296	<0.001	1.345	1.332	1.358
Medicare vs Self-Pay	0.618	<0.001	1.856	1.821	1.892
Medicare vs No Charge	0.247	<0.001	1.280	1.193	1.374
Medicare vs Other Insurance	0.781	<0.001	2.184	2.149	2.219
RACE					
Black v White	0.118	<0.001	1.125	1.115	1.136
Hispanic v White	-0.007	0.228	0.993	0.981	1.005
Asian v White	0.118	<0.001	1.125	1.104	1.147
Native American v White	0.237	<0.001	1.267	1.219	1.318
Other Races v White	0.242	<0.001	1.273	1.250	1.297
REGION					
NE vs MW	-0.207	<0.001	0.813	0.805	0.821
NE vs S	-0.037	<0.001	0.963	0.955	0.972
NE vs W	-0.091	<0.001	0.913	0.904	0.922
CCI	0.049	<0.001	1.050	1.046	1.054
Alzheimer's	-0.591	<0.001	0.554	0.543	0.565
CHF	0.281	<0.001	1.325	1.313	1.336
Colon Cancer	0.136	<0.001	1.145	1.100	1.193
COPD	-0.014	0.003	0.987	0.978	0.995
CVD	0.512	<0.001	1.669	1.635	1.704
Senile degeneration of the brain	0.442	0.003	1.556	1.158	2.092
ESRD	0.838	<0.001	2.313	2.284	2.343
Lung Cancer	0.379	<0.001	1.461	1.428	1.495
Pancreatic Cancer	0.071	<0.001	1.074	1.035	1.115
Parkinson's Disease	-0.411	<0.001	0.663	0.647	0.679
Prostate Cancer	-0.317	<0.001	0.729	0.706	0.752
Sepsis	1.481	<0.001	4.397	4.365	4.429
Palliative Care/DNR					
No PC/DNR vs PC Only	3.578	<0.001	35.795	35.387	36.207
No PC/DNR vs DNROnly	2.279	<0.001	9.763	9.676	9.851
No PC/DNR vs PC and DNR	4.104	<0.001	60.592	60.078	61.111
Constant	-5.998	<0.001	0.002		

Outcome LOS (Log LOS)

Linear Regression	Unstandardized Coefficients		Standardized Coefficients	t	Sig.
**Outcome = LOG_LOS**	**B**	**Std. Error**	**Beta**		
YEAR					
2016 v 2017	-0.020	0.000	-0.011	-44.412	<0.001
2016 v 2018	-0.022	0.000	-0.012	-48.261	<0.001
2016 v 2019	-0.021	0.000	-0.011	-45.764	<0.001
AGE	0.003	0.000	0.074	157.195	<0.001
Gender - Male	0.079	0.000	0.049	237.505	<0.001
Insurance					
Medicare vs Medicaid	0.014	0.001	0.007	24.095	<0.001
Medicare vs Private	-0.100	0.000	-0.055	-203.083	<0.001
Medicare vs Self-Pay	-0.082	0.001	-0.020	-90.156	<0.001
Medicare vs No Charge	-0.043	0.003	-0.003	-15.521	<0.001
Medicare vs Other Insurance	-0.058	0.001	-0.012	-56.518	<0.001
RACE					
Black v White	0.094	0.000	0.043	200.396	<0.001
Hispanic v White	-0.005	0.001	-0.002	-9.023	<0.001
Asian v White	0.033	0.001	0.007	32.799	<0.001
Native American v White	0.059	0.002	0.006	29.329	<0.001
Other Races v White	0.039	0.001	0.008	41.237	<0.001
REGION					
NE vs MW	-0.076	0.001	-0.039	-150.505	<0.001
NE vs S	-0.049	0.000	-0.030	-107.723	<0.001
NE vs W	-0.123	0.001	-0.061	-233.354	<0.001
CCI	0.001	0.000	0.003	6.147	<0.001
Alzheimer's	0.115	0.001	0.016	78.508	<0.001
CHF	0.165	0.001	0.069	295.740	<0.001
Colon Cancer	0.131	0.003	0.008	37.556	<0.001
COPD	0.082	0.001	0.036	157.604	<0.001
CVD	0.054	0.002	0.007	35.488	<0.001
Senile d of the brain	0.028	0.036	0.000	0.769	0.442
ESRD	0.236	0.001	0.054	263.712	<0.001
Lung Cancer	0.047	0.002	0.004	21.065	<0.001
Pancreatic Cancer	0.089	0.004	0.005	25.080	<0.001
Parkinson's Disease	0.096	0.002	0.013	63.663	<0.001
Prostate Cancer	-0.206	0.002	-0.021	-103.873	<0.001
Sepsis	0.398	0.001	0.123	603.961	<0.001
Palliative Care/DNR					
No PC/DNR vs PC Only	0.423	0.002	0.050	248.544	<0.001
No PC/DNR vs DNROnly	0.118	0.001	0.032	155.033	<0.001
No PC/DNR vs PC and DNR	0.246	0.001	0.044	212.271	<0.001
Constant	1.013	0.001		956.965	<0.001

Outcome Charges (LOG_Charges)

**Linear Regression**	**Unstandardized Coefficients**		**Standardized Coefficients**	**t**	**Sig.**
**Outcome = LOG_CHG**	**B**	**Std. Error**	**Beta**		
YEAR					
2016 v 2017	0.037	0.001	0.017	68.762	<0.001
2016 v 2018	0.083	0.001	0.037	153.643	<0.001
2016 v 2019	0.145	0.001	0.065	267.365	<0.001
AGE	0.011	0.000	0.237	516.405	<0.001
Gender - Male	0.155	0.000	0.079	393.803	<0.001
Insurance					
Medicare vs Medicaid	-0.054	0.001	-0.022	-78.672	<0.001
Medicare vs Private	0.071	0.001	0.032	122.044	<0.001
Medicare vs Self-Pay	-0.016	0.001	-0.003	-15.023	<0.001
Medicare vs No Charge	0.129	0.003	0.008	40.066	<0.001
Medicare vs Other Insurance	-0.012	0.001	-0.002	-10.054	<0.001
RACE					
Black v White	0.064	0.001	0.024	114.108	<0.001
Hispanic v White	0.131	0.001	0.043	203.492	<0.001
Asian v White	0.056	0.001	0.009	46.901	<0.001
Native American v White	-0.148	0.002	-0.012	-61.919	<0.001
Other Races v White	0.147	0.001	0.026	129.292	<0.001
REGION					
NE vs MW	-0.173	0.001	-0.073	-287.328	<0.001
NE vs S	-0.027	0.001	-0.014	-51.018	<0.001
NE vs W	0.290	0.001	0.118	462.946	<0.001
CCI	-0.020	0.000	-0.041	-84.514	
Alzheimer's	-0.212	0.002	-0.024	-121.915	<0.001
CHF	0.110	0.001	0.038	165.670	<0.001
Colon Cancer	0.014	0.004	0.001	3.434	0.001
COPD	0.034	0.001	0.012	54.821	<0.001
CVD	0.157	0.002	0.017	86.745	<0.001
Senile degeneration of the brain	-0.761	0.043	-0.004	-17.849	<0.001
ESRD	0.332	0.001	0.063	312.970	<0.001
Lung Cancer	-0.012	0.003	-0.001	-4.622	<0.001
Pancreatic Cancer	-0.021	0.004	-0.001	-5.064	<0.001
Parkinson's Disease	-0.076	0.002	-0.008	-42.391	<0.001
Prostate Cancer	0.015	0.002	0.001	6.342	<0.001
Sepsis	0.357	0.001	0.091	454.809	<0.001
Palliative Care/DNR					<0.001
No PC/DNR vs PC Only	0.186	0.002	0.018	92.610	<0.001
No PC/DNR vs DNROnly	-0.149	0.001	-0.034	-164.628	<0.001
No PC/DNR vs PC and DNR	0.039	0.001	0.006	28.414	<0.001
Constant	9.572	0.001		7618.676	<0.001
